# The analysis of dissolved inorganic carbon in liquid using a microfluidic conductivity sensor with membrane separation of CO_2_

**DOI:** 10.1007/s10404-020-02339-1

**Published:** 2020-04-25

**Authors:** M. Tweedie, D. Sun, D. R. Gajula, B. Ward, P. D. Maguire

**Affiliations:** 1 Nanotechnology and Integrated BioEngineering Centre (NIBEC), Ulster University, Jordanstown, Newtownabbey, BT37 0QB UK; 2 School of Mechanical and Aerospace Engineering, Queen’s University, Belfast, BT9 5AH UK; 3 Department of Electrical and Computer Engineering, Clemson University, Clemson, SC 29634 USA; 4 AirSea Laboratory, Ryan Institute and School of Physics, National University of Ireland, Galway, Ireland

**Keywords:** Dissolved inorganic carbon, CO_2_, Conductivity, Impedance, Microfluidics, PDMS membrane

## Abstract

Autonomous continuous analysis of oceanic dissolved inorganic carbon (DIC) concentration with depth is of great significance with regard to ocean acidification and climate change. However, miniaturisation of in situ analysis systems is hampered by the size, cost and power requirements of traditional optical instrumentation. Here, we report a low-cost microfluidic alternative based on CO_2_ separation and conductance measurements that could lead to integrated lab-on-chip systems for ocean float deployment, or for moored or autonomous surface vehicle applications. Conductimetric determination of concentration, in the seawater range of 1000–3000 µmol kg^−1^, has been achieved using a microfluidic thin-film electrode conductivity cell and a membrane-based gas exchange cell. Sample acidification released CO_2_ through the membrane, reacting in a NaOH carrier, later drawn through a sub-µL conductivity cell, for impedance versus time measurements. Precision values (relative standard deviations) were ~ 0.2% for peak height measurements at 2000 µmol kg^−1^. Comparable precision values of ~ 0.25% were obtained using a C4D electrophoresis headstage with similar measurement volume. The required total sample and reagent volumes were ~ 500 µL for the low volume planar membrane gas exchange cell. In contrast, previous conductivity-based DIC analysis systems required total volumes between 5000 and 10,000 µL. Long membrane tubes and macroscopic wire electrodes were avoided by incorporating a planar membrane (PDMS) in the gas exchange cell, and by sputter deposition of Ti/Au electrodes directly onto a thermoplastic (PMMA) manifold. Future performance improvements will address membrane chemical and mechanical stability, further volume reduction, and component integration into a single manifold.

## Introduction

Atmospheric carbon dioxide concentration has increased significantly due to human activity and is a primary contributor to global warming. The ocean is a major sink for anthropogenic CO_2_, mitigating the effects of atmospheric emissions with the CO_2_ uptake causing changes to ocean chemistry (Sabine et al. 2004). However, the ability of the oceans to continue absorption at historic levels is uncertain, particularly since, in the first instance, CO_2_ solubility decreases with rising temperature. To permit modelling of CO_2_ atmosphere:ocean interchange and CO_2_ ocean absorption with climate change, depth profile measurements are required continuously from across the world’s oceans. While the importance of ocean chemistry is recognised, our ability to measure ocean variables with sufficient resolution and accuracy is severely restricted due to a lack of analysis systems capable of low-cost and widespread deployment.

The state of the oceanic CO_2_ system can be determined by measuring at least two of four variables, namely, CO_2_ partial pressure (pCO_2_), pH, the total alkalinity (*A*_T_), and the dissolved inorganic carbon (DIC) (Millero 2007). The latter consists of CO_2_ dissolved in seawater, mainly as bicarbonate, HCO_3_^−^, and carbonate CO_3_^2−^ ions, with small amounts of carbonic acid, H_2_CO_3_, and CO_2_ molecules. The standard concentration of dissolved CO_2_ in seawater at sea level is around 1900 µmol kg^−1^, rising to > 2400 µmol kg^−1^ at depth (Wang et al. 2013), assuming current values of pH (~ 8.2) and atmospheric CO_2_ content (400 ppm) (Dlugokencky and Tans 2019). The relevant ions cannot be detected directly against the large background of seawater ions, which are mostly from dissolved NaCl and MgSO_4_, with typically 35 g of salts per litre. Therefore, the DIC ions must be first extracted from the seawater by conversion to CO_2_ gas molecules, for transfer through a gas permeable membrane into a receiving channel for measurement. The addition of excess acid to seawater, reducing the pH to ≤ 4.5, releases DIC as gaseous CO_2_ into the aqueous phase for membrane separation. The receiving channel may comprise vacuum, inert gas, or reactive solution, where the DIC amount transferred can be measured by a variety of methods.

At present, CO_2_ measurements are typically carried out by coulometry using large-volume sensors on research ships, according to Standard Operating Protocols (Dickson et al. 2007). Although both pCO_2_ and pH are very sensitive to temperature and pressure, they can be measured away from sampling depths under carefully controlled conditions, and while microfluidic miniaturisation approaches for in situ surface measurements are currently being developed, complementary autonomous measurements of DIC or *A*_T_ would be preferred (Rérolle et al. 2018; Clarke et al. 2017). Given the technical challenges, however, such approaches have yet to be realised (Byrne 2014).

Non-conductimetric measurement methods for DIC determination have been reported, using e.g. gas chromatography (Hansen et al. 2013), and membrane inlet mass spectroscopy (Tortell 2005; Guéguen and Tortell 2008; Bell et al. 2011; Freije-Carrelo et al. 2018). Optical methods include non-dispersive IR (Kaltin et al. 2005; Fassbender et al. 2015; Bass et al. 2012a, b) and spectrophotometry (Nakano et al. 2006; Wang et al. 2007, 2013; Liu et al. 2013). The partial pressure of CO_2_, (pCO_2_), has also been measured by spectrophotometry, on an autonomous moored submersible instrument (DeGrandpre et al. 1995). Recently, a compact LED-based spectrophotometric instrument for in situ measurement has been reported, capable of achieving DIC precision of ± 2.5 μmol kg^−1^ with a 700 µL sample cell and total sample and reagent volumes of 9000 µL per sample (Wang et al. 2015). However, these are all large systems and unfortunately do not offer an obvious route to miniaturisation.

Coulometry is the recommended standard operating procedure for bench DIC determination (Dickson et al. 2007). However, a flow injection method (Hall and Aller 1992) based on a conductimetric technique, with microfluidic channels on either side of a planar gas exchange membrane, offers greater opportunity for miniaturisation. Here, the CO_2_ is collected in an alkaline receiving solution, typically NaOH. Provided the pH of this solution is sufficiently low, the added CO_2_^aq.^ is converted to CO_3_^2−^ through reaction with OH^−^ and due to the lower mobility of the former, the fluid conductivity is reduced in direct proportion to the added CO_2_ concentration. The use of conductimetric techniques for determining DIC concentration has also been reported more recently (Sayles and Eck 2009; Bresnahan and Martz 2018). Fluid conductance/impedance techniques are rarely considered for high-resolution chemical analysis as accuracy and precision are generally poorer than with colorimetric or optical techniques. This is exacerbated with small sample volumes when deployed in a microfluidic structure. Nevertheless, in this case, because of the high potential for microfluidic system miniaturisation, we examine the applicability of these techniques to DIC measurements.

Sayles and Eck suggest that the optimum reference receiver solution concentration for ~ 2 mM DIC measurement is ~ 7 mM NaOH, but Hall and Aller originally achieved acceptable results for 10 mM NaOH. The receiving solution conductivity is ~ 170 mS m^−1^ for 7 mM, and 220 mS m^−1^ for 10 mM NaOH. Sayles and Eck used a ~ 150 µL conductivity cell containing Pt wires and a 1000 µL sample cell, with a silicone inner tube containing NaOH which acts as the membrane receiving cell (330 µL) (Sayles and Eck 2009). The analysis time per sample was ~ 1 h and required sample and reagent volumes (acid, NaOH) of 2500 µL and 8500 µL, respectively. In laboratory and field tests, they obtained precision values of ± 2.2 µmol kg^−1^ and ± 3.6 µmol kg^−1^, equivalent to relative standard deviations (RSD) of ± 0.11% and ± 0.18%, respectively. For comparison, laboratory DIC coulometric calibration measurements using standard operating procedures, have cited precision values of ± 1.5 to ± 2.0 µmol kg^−1^ (Dickson et al. 2007).

Plant et al. (2009) also used a conductimetric approach to detect ammonium in marine environments. The sample and receiving channels (100 µL/50 µL) were formed in two thermoplastic blocks which sandwiched a thin PTFE membrane and the conductivity cell (50 µL) comprised two macroscopic plates. A detection limit of 0.2 µM was achieved with minimum error levels of 2.5% (above 3 µM). The accuracy was limited by the transfer of ions across the semi-porous PTFE membrane. The analysis time was ~ 7 min and required sample and reagent volumes of 1650 µL and 3800 µL, respectively.

Other conductimetric cell designs include concentric metal tubes (Henríquez et al. 2014) and contactless arrangements involving insulated wire pairs (Hoherčáková and Opekar 2005) or a capillary electrophoresis (C4D) headstage (Bresnahan and Martz 2018). In the latter work, diffusion time, membrane type and surface area to cell volume was investigated for sample volumes up to 300 µL. Although the headstage unit was relatively large, each measurement point required a very small volume of liquid (< 5 µL) and with signal averaging, a precision of ~ 0.1% was achieved.

One possible application of a miniaturized DIC measurement approach is integration onto profiling float platforms for open ocean monitoring of the upper 2000 m (Bresnahan et al. 2017). DIC insensitivity to pressure and temperature would allow for sample collection at different depths for subsequent analysis elsewhere. This could include use at a preset park depth, or even on-ship or in a laboratory, depending on the degree of miniaturisation achievable for the whole system. This would require detailed design of reagent storage volumes, on-chip multiple sample storage, and pumps and valves dimensions.

The Argo network is a well-known ocean profiling system currently consisting of > 3000 globally distributed autonomous ocean profile floats, providing continuous depth profiling of salinity and temperature (Roemmich et al. 2009). These are intended to drift in the open ocean for at least 5 years, early failures notwithstanding, and are programmed to profile the upper 2 km of ocean every 5–10 days with the data then transmitted to satellite. Typically, these floats, for diameters greater than 170 mm, can offer a sensor payload capability of at least 1.5 kg, while currently available sensors vary in volume from about 0.2 L to over 3 L. On this platform, DIC sensors would need to operate long term with total reagent volumes preferably less than 1 L. At 100 samples per profile, reagent limits per sample are, therefore, < 100 µL and with sample flushing between measurements, this may be lowered considerably. Overall, this small cell volume requirement represents a significant challenge for both fabrication and detection accuracy and precision. On a less restrictive platform, of course, such as for instruments tethered to moored buoys, or in autonomous surface vehicles, where volume is not so restricted, the DIC sensors reported here will be more easily implemented.

Other constraints on DIC sensors on oceanic probes relate to the long-term and harsh environment operation, where robust chemically resilient device bonding is essential with additional features, e.g. multiple layers, mixed materials and multi-channel structures, not normally considered in mainstream microfluidics which is focussed primarily on biomedical applications. Recently, though, we demonstrated long-term PDMS membrane bonding within a thermoplastic manifold (Tweedie et al. 2019) as well high-pressure resilient thermoplastic bonding for multi-layer and multi-channel devices (Sun et al. 2015).

In this work, we investigate direct contact conductivity cells with active volumes between 0.5 and 2.0 µL for DIC detection in the seawater range 1900–2400 µmol kg^−1^. For these volumes, the use of macroscopic wires or plates as electrodes is not feasible and instead, we developed a thin-film metallisation process onto PMMA using a sputtered thin film of gold (< 200 nm) on top of a 10 nm adhesion promoting inter layer of sputtered Ti.

## Materials and experimental methods

### Fabrication

Various components were fabricated in NIBEC (Nanotechnology and Integrated Bio-Engineering Centre) to facilitate this research. First, a planar Au electrode microfluidic conductivity cell was required for electrical measurements. Second, a membrane exchange diffusion cell, with a gas permeable membrane, was needed for transferring liberated CO_2_ from the acidified sample solution into NaOH receiving solution for DIC determination. The sample solution was prepared at various molarities directly related to the known DIC range in seawater, from NaHCO_3_ stock solution (Sigma Aldrich). Third, an asymmetric Y meter was required for accurate dynamic metering of acid into sample solution, for automated DIC measurements.

#### Conductivity cell

**Fig. 1:**
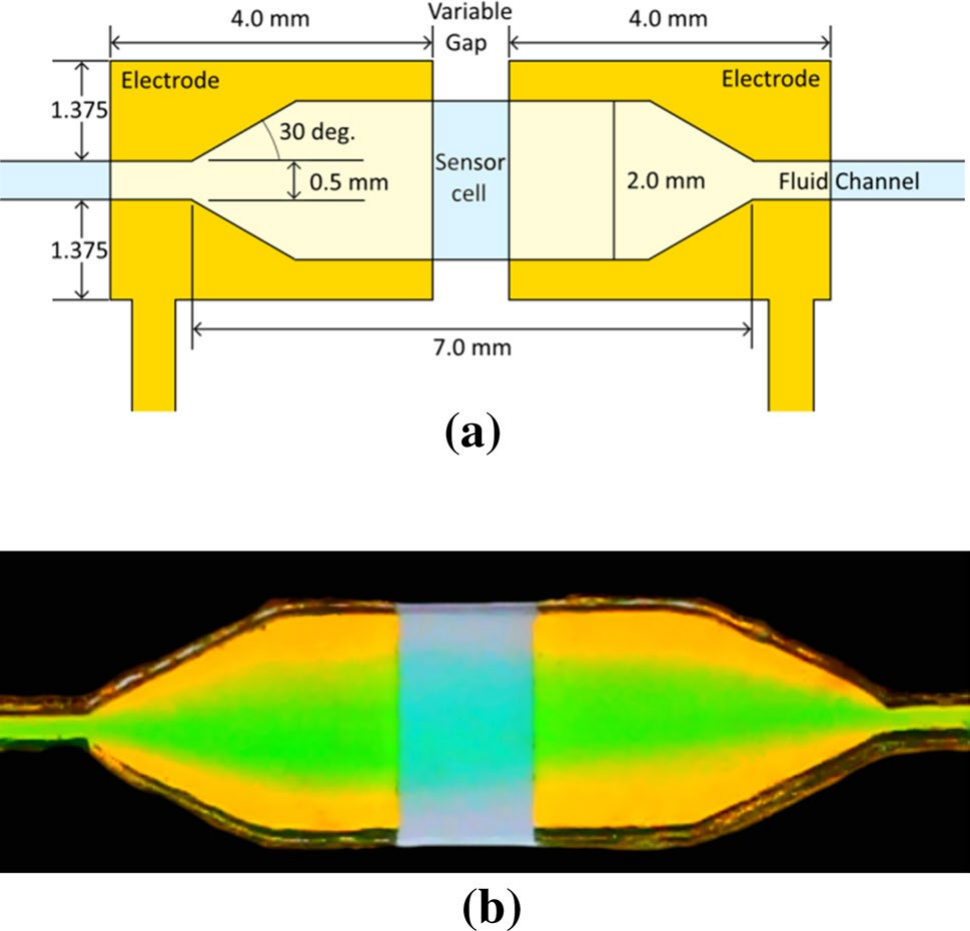
**a** Electrode pair and sensor cell schematic. **b** Photo of Au/Ti electrode pair and sensor cell with green dye input, through DI water prefill. A modest excess flow volume is required to totally clear the cell of previous sample contents. Dimensions are as in (**a**)

Metal electrodes were fabricated by RF sputtering of a Ti adhesion layer (< 10 nm), followed by DC magnetron sputtering of an Au layer (~ 200 nm) through a shadow mask onto flat PMMA substrates to form the electrode pattern. This was then bonded to a second PMMA manifold, wherein the fluid channel had been formed, using CHCl_3_ solvent vapour-assisted thermocompression bonding at 80 °C in vacuum (Sun et al. 2015). Electrode integrity was tested for resilience against NaOH (9 mM, pH 12) corrosion over several weeks. The electrode cell construction is illustrated in Fig. 1a. Microfluidic channels were milled with an equal height and width of 0.5 mm and the electrode gap was varied from 0.25 to 2 mm, giving an active measurement volume range of 0.5–2.0 µL. The cells were completed with the attachment of Nanoport fluidic connections using epoxy and sealed with silicone, for subsequent immersion in a temperature-controlled (± 0.05 °C) water bath. Green dye flow through a DI water filled sensor cell is shown in Fig. 1b, where some excess solution is required to fully remove previous sample contents.

#### Membrane exchange diffusion cell

**Fig. 2:**
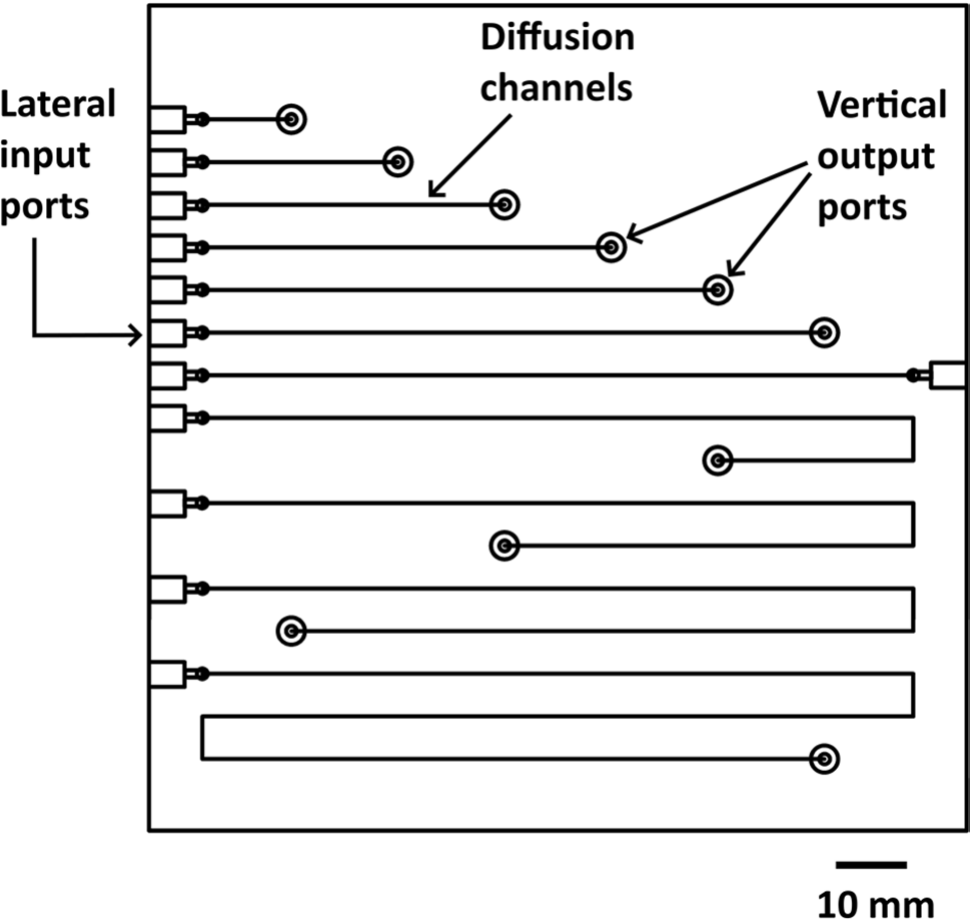
Plan view drawing of multiple membrane exchange diffusion cells, where the PDMS membrane is sealed between opposite halves

To operate at reduced system volumes, the use of silicone tubes as previously reported may not be practical for gas exchange diffusion cells (Nakano et al. 2006; Wang et al. 2013, 2015; Liu et al. 2013; Bresnahan and Martz 2018). Here, though, we used a membrane exchange diffusion cell consisting of two PMMA substrates with patterned sample and receiving chambers along with associated microfluidic channels and fluid connectors, separated by a thin membrane sheet. Recently, we investigated a range of membrane sheet materials to determine optimum CO_2_ exchange versus unwanted ion conductivity and from this it was clear that PDMS performance is far superior to other materials (Tweedie et al. 2019), especially compared to PTFE membranes that have been previously reported (Plant et al. 2009). Here, we have used a series of chambers of 1 mm^2^ cross-section and variable length from ~ 12 to 300 mm, CNC micromachined on separate substrates with a 50 μm PDMS film membrane (Goodfellow Cambridge Ltd.) fixed between the two opposing sides, see plan view, Fig. 2.

#### Y meter fabrication

**Fig. 3:**
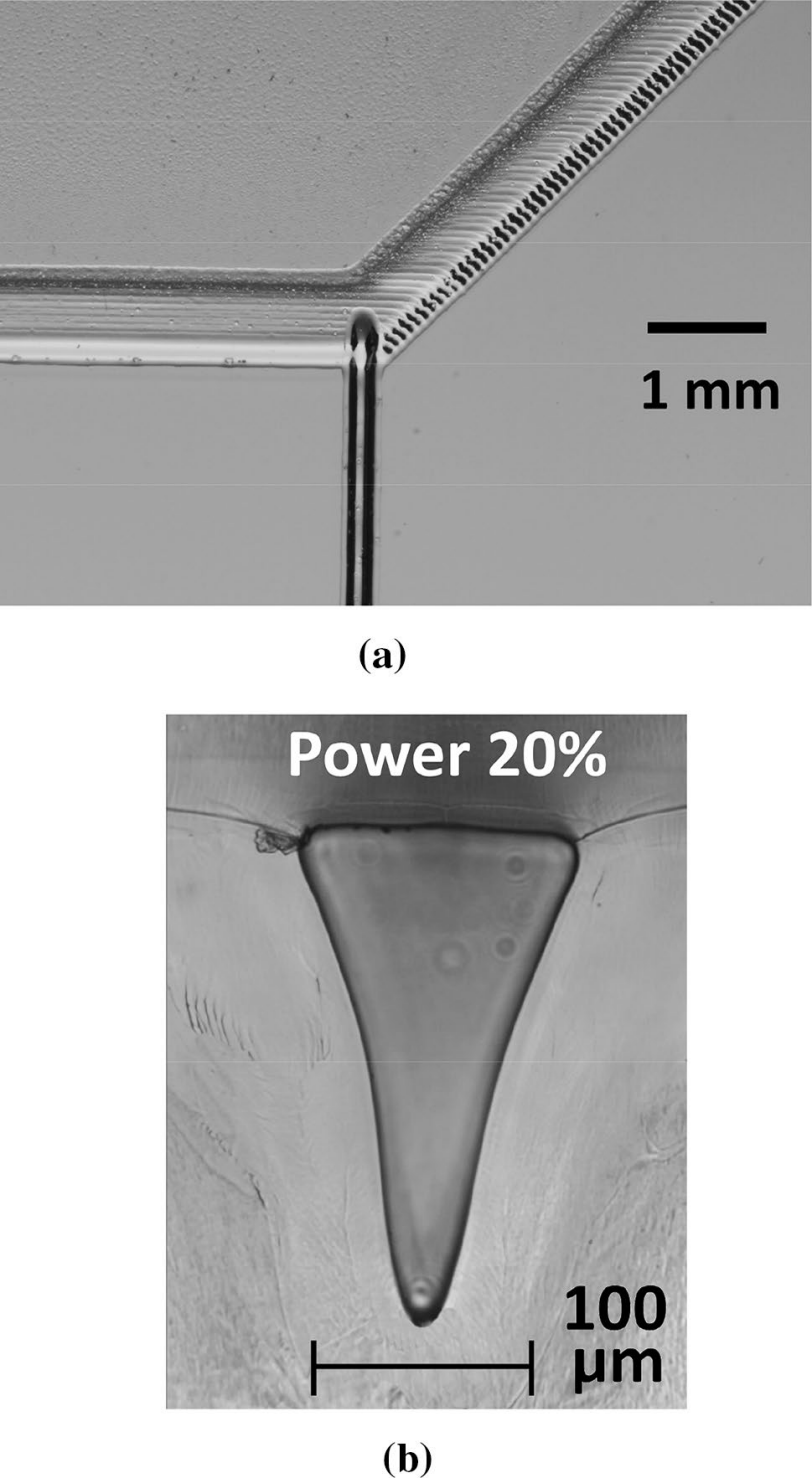
**a** Asymmetric Y meter example, as engraved by CO_2_ laser. The thin vector engraved channel is vertical in the image plane, while the remainder is engraved by raster scan from left to right. Raster engraved at 8.75 W, vector at 5 W, both at 0.3 m/s. The rastered channels are wider here, than in practice, for better visibility. Actual bonded devices used a reduced NaHCO_3_ and output channel width, compared to this example, of ~ 400 µm. **b** Example of vector engraved channel cross-section in PMMA, post bond. Engraving power 5 W, at speed of 0.3 m/s

For dynamic DIC mixing, HCl acid is injected into the NaHCO_3_ flow stream, rather than by bulk pre-mixing. For this, we used an asymmetric Y-junction reagent meter, fabricated in-house by CO_2_ laser etching of PMMA (Fig. 3a) and sealed by solvent (CHCl_3_) vapour-assisted bonding (Sun et al. 2015), as in the example in Fig. 3b. The laser used was a 25 W VLS2.30 model from Universal Laser Systems. The NaHCO_3_ input channel width was ~ 400 µm, and depth was ~ 298 µm, written in raster mode at 35% power (8.75 W), and 0.3 m/s. The base of the channel was approximately flat over a width of ~ 100 µm. A set of vectors, written at powers of 12.5, 15 and 20% levels, and speeds of 0.3 m/s, was used to produce narrow HCl input channels, giving Y meters of meter ratios ~ 14:1, 10:1 and 6:1. These devices have been separately described, along with various long channel restrictors, for controlling metering ratios (Tweedie et al. 2020). The dimensions of the vectored channel for the 6:1 Y meter are ~ 130 µm top width, and depth ~ 230 µm, with a roughly V-shaped cross-sectional profile, Fig. 3b. This device was used in these tests, with 0.1 M HCl input, effectively diluted down 6X by the Y meter to ~ 16.7 mM, which is sufficient excess to liberate all CO_2_ from the 2 mM NaHCO_3_ source.

### Experimental methods

A Solartron SI 1260 Impedance/Gain-phase analyser was used, with SI 1287 or SI 1294 impedance interfaces, to measure the fluid impedance and phase angle at data acquisition rates from 1 per minute to a maximum 1 per 5 s (0.2 Hz). The excitation frequency was chosen to minimise the absolute value of phase angle, so that readings had a minimum capacitive component and maximum resistive component. Impedance measurement software was either SMaRT, which, for impedance terms, outputs the magnitude, |Z|, or ZView, which recorded both the real and imaginary components, Z' and Z’’, where Z’ equates to the resistance. Cell calibration was undertaken with standard KCl solutions (Hanna Instruments).

For initial sensor testing, |Z| was measured for 9 mM NaOH over several hours to assess thermal drift, and sensor noise. Then two solutions with a conductivity difference of 1% (139.9 mS m^−1^ and 141.3 mS m^−1^) were used for repetitively switched sampling. An Elveflow AF1 P1600 pump, with manually operated valves, was used to pressurise a bottle of each solution in turn and the solution bottles and sensor cell were held in a Heraeus oven at 27.5 °C.

Before the asymmetric Y meter had been fabricated, DIC solutions were made via bulk premix of acid and sample. Here, CO_2_ loaded solutions were prepared by mixing preset amounts of 0.1 M NaHCO_3_ (Sigma Aldrich UK) with degassed DI water, creating a DIC content from 1.0 to 3.0 mM, depending on dilution ratio. HCl (0.1 M) was then mixed with this in a 5% *v*/*v* (acid:sample) ratio, either in a sealed vessel, or in a syringe. The acid was effectively diluted to ~ 5.0 mM, of sufficient excess to liberate the maximum of 3.0 mM CO_2_ from the DIC solution. After 20–25 min reaction time, the sample was injected at the diffusion test cell input, under positive pressure. For initial diffusion cell comparisons and calibration testing, the receiver (NaOH) chamber was purged with fresh solution using a programmable syringe pump (Aladdin) before the CO_2_ sample was added to the sample chamber. A diffusion time of 20 min allowed sufficient exchange of CO_2_ from sample to receiver chamber across the membrane. The CO_2_-loaded NaOH solution was then pumped through the Au sensor cells, for impedance measurement, and the cycle repeated as desired. Calibrations were performed for 1.0–3.0 mM DIC using a ~ 200 μL receiving chamber and a total sample volume of 1 mL.

Subsequently, once a calibration had been achieved using bulk pre-mixing of acid and sample, experiments proceeded to use dynamic acid and sample mixing via the fabricated asymmetric Y meter, with measurements using the Au sensor. For comparison, a contactless capillary electrophoresis headstage and controller (eDAQ ET125/ER225 C4D) was also used (Bresnahan and Martz 2018). The fluid conductivity is determined within a capillary tube using external electrodes driven at high frequency (1.2 MHz) and voltage (200 V), acquiring data points at a rate of 1 Hz. The active eDAQ C4D measurement volume between electrodes was ~ 2 μL.

**Fig. 4:**
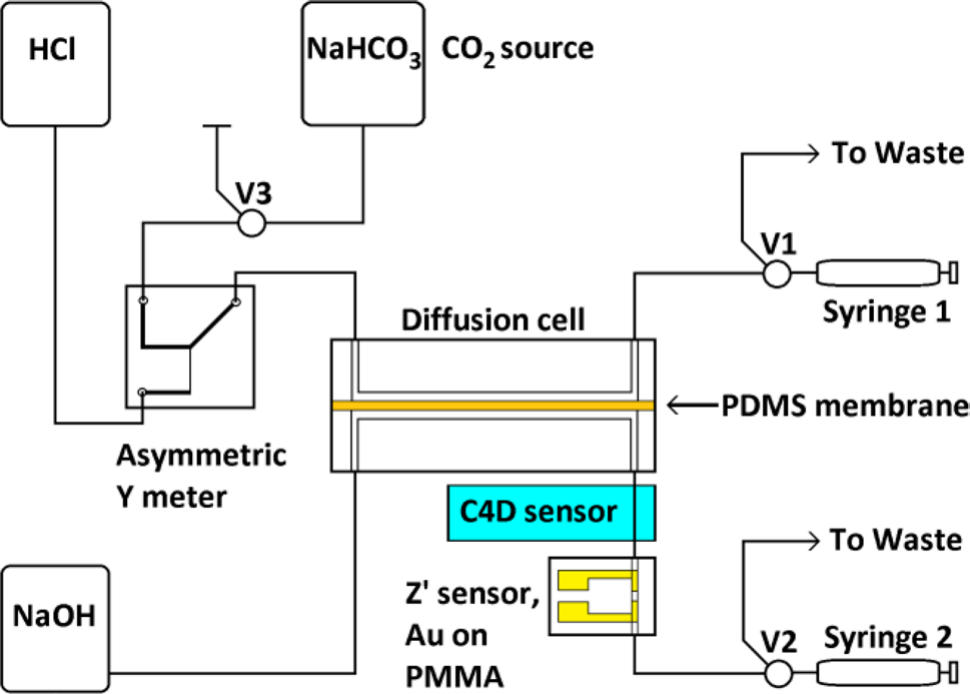
Schematic diagram of negative pressure system for Z’ and C4D measurements of DIC. The temperature stabilised water bath is omitted for clarity. The three port valves are V1–V3

Exchange cell volumes were reduced to 100 μL, with NaOH reagent volumes for the elution curve of 350–600 μL, depending on flow rate. Cetoni NeMeSys Low Pressure syringe pumps, with QMix control software, were used to draw fluid through both sides of the gas exchange cell in sequence. The DIC solution was drawn through at a fixed rate (7.5–8.5 μL s^−1^), followed by 15 min diffusion. NaOH was then drawn through at a fixed rate 1.5–1.7 μL s^−1^. Each elution peak was therefore at least 200 s. A small number of samples were drawn through at 12 μL s^−1^ to evaluate the impact of high flow rates. A temperature stabilised water bath, using an NE4-D/CT Clifton digital temperature-controlled heater and stirrer, in combination with a DC1-300 Clifton chiller, was used to compensate for long-term thermal drifts. The stated temperature sensitivity, after stabilisation, is ± 0.05 °C. The C4D sensor head was immersed in a silicone oil bath (Xiameter PMX200 silicone fluid, 20cS) within the water bath, while the Au sensor microfluidics were immersed directly in the water bath, which was set at 25 °C. The reagent bags were not temperature stabilised. A schematic diagram of the negative pressure experimental setup is shown in Fig. 4.

## Results and discussion

**Fig. 5:**
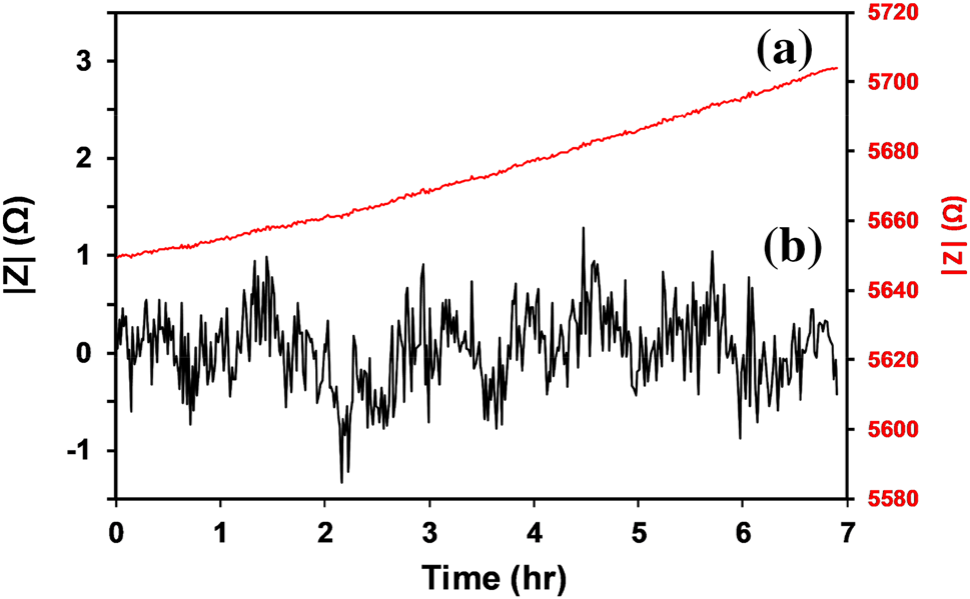
Time variation in |Z| for 9.1 mM NaOH solution (~ 200 mS m^−1^) for **a** raw data, and **b** drift-corrected data, using a fourth order polynomial fit. The drift-corrected data gives an rms noise of 0.39 Ω

Optimum test conditions for impedance measurements were determined initially using a 9.1 mM (~ 200 mS m^−1^) NaOH solution. The phase angle reduced to a minimum absolute value at ~ 63.096 kHz, for 0.5 mm gap electrodes, and at 50–100 kHz for 0.9 mm gap electrodes. The optimum signal-to-noise ratio (SNR) occurred at a software selected level of 100 mV rms. Continuous |Z| impedance measurements were acquired at 60 s intervals over 7 h, as shown in Fig. 5. The expected temperature dependence of ionic liquid conductivity is ~ 2%/°C, while the |Z| drift in Fig. 5 is ~ 0.95%, as would be produced by a temperature drift of 0.475 °C. A fourth order polynomial was fitted to the data to subtract the background drift, for the purpose of evaluating the rms noise, as shown in Fig. 5. The rms noise for 9.1 mM (~ 200 mS m^−1^) NaOH solution is 0.39 Ω. The estimated cell constant is ~ 1140 m^−1^.

**Fig. 6:**
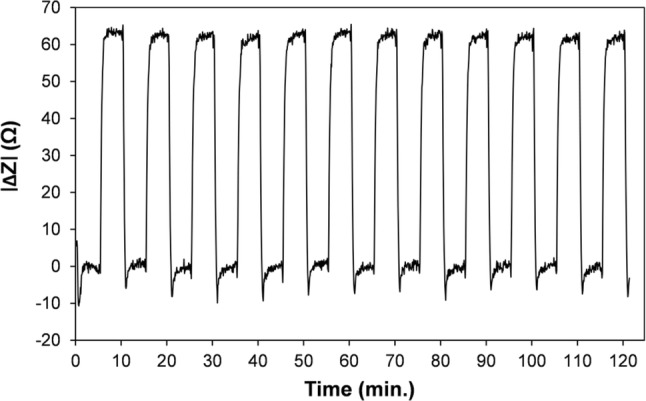
Repeatability data for switching between two fluids with a conductivity difference of 1% (139.9 mS m^−1^ and 141.3 mS m^−1^)

The repeatability of the measurement was examined by cyclic switching between two KCl solutions with a nominal conductance difference of 1%. Background drift correction, similar to that in Fig. 5, was applied, see Fig. 6. The average impedance was 8522 Ω, and average |Z| was calculated from 25 data points sampled, just prior to each switching event. The |Z| time series is shown in Fig. 6 (63,096 Hz, 100 mV rms input, and 0 V DC), where the average |Z| is ~ 62.5 Ω and the estimated rms noise is ~ 0.71 Ω. The rms sensor noise is at a very low level compared to the background (~ 0.008%), but the precision for DIC measurement will depend firstly on the |Z| signal change produced by the maximum CO_2_ level to be detected. The rms noise divided by the maximum signal, expressed as a percentage, can indicate the best theoretical precision achievable for the sensor noise component alone, ignoring other noise contributors for now. Known full system spectrophotometric and conductimetric field precision values (RSD) lie in the range of 0.14–0.18% (Wang et al 2013, 2015; Sayles and Eck 2009), equating to ~ 2.8–3.6 µmol kg^−1^, for an average DIC level of 2000 µmol kg^−1^. The final system precision will be worse than the sensor precision because of factors such as measurement fluctuations from e.g. membrane relaxations, residual temperature drifts, and long-term reagent changes.

**Fig. 7:**
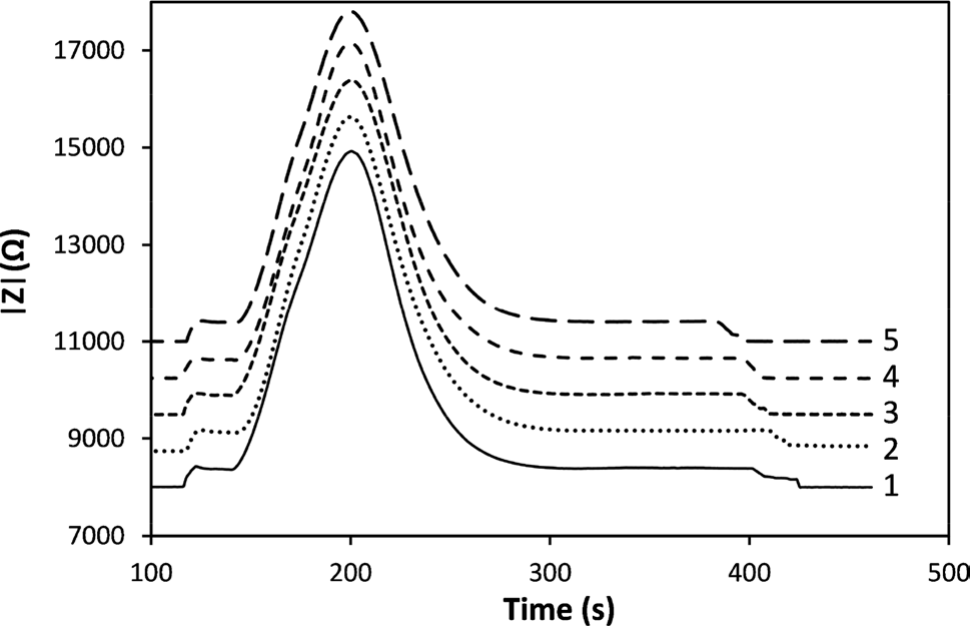
|Z| for repeat samples of 2 mM TCO_2_ using a 0.9 mm electrode gap, 100 mV rms, 0 V DC, 100 kHz, with 20 min diffusion

For DIC calibration testing, under acid:sample DIC premix conditions, 200 µL sample and receive chambers were used with a 50 μm PDMS membrane. Diffusion tests were carried out for 2000 µM DIC and 0.9 mm electrode gap, at 100 kHz and 100 mV rms. Each elution peak was ~ 120 s with peak heights of ~ 6600 Ω, after background subtraction. The measured rms noise was 1.05 Ω, giving a SNR of ~ 6285:1. Although the measured SNR suggests a possible theoretical precision of 0.016%, the observed rms peak variation, after background subtraction, in Fig. 7 is actually ~ 0.7%, as a result of uncorrected background ripples, which are suspected to be partly due to fluctuations in the flexible PDMS membrane, from residual flow perturbations. Furthermore, these initial experiments were not highly temperature stabilised, so greater variability would be expected from this factor also.

**Fig. 8:**
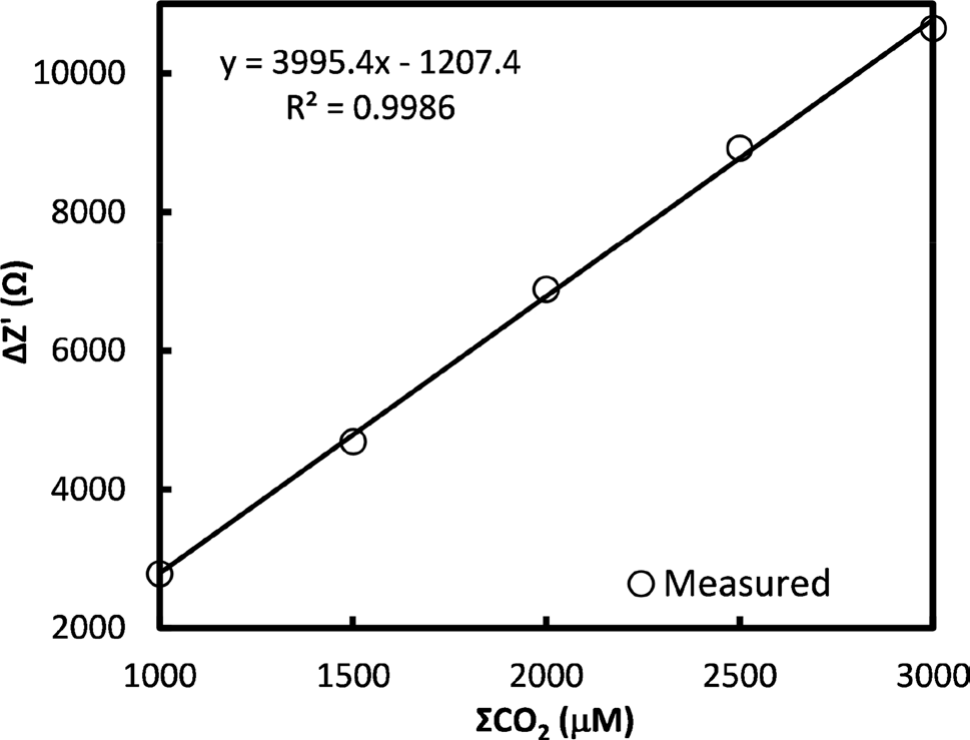
DIC calibration for 200 µL sample and receive chambers and 20 min diffusion (0.9 mm gap, 100 mV rms, 0 V DC, 100 kHz)

The calibration curve for 1000–3000 µM DIC is given in Fig. 8 and is observed to be linear (*R*^2^ > 0.99) over this range. The RSD (not shown) is less than 0.8%.

**Fig. 9:**
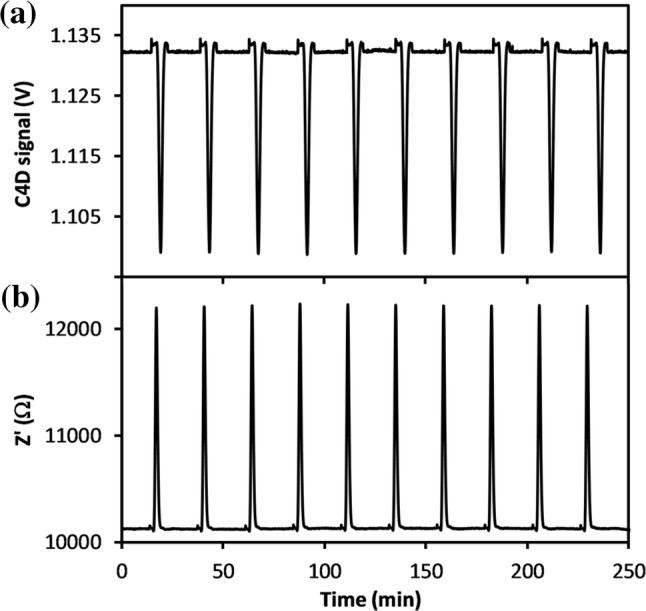
**a** C4D peak sequence sample with 2000 µM DIC solution, a 100 µL channel and diffusion time of 15 min, **b** Z’ peak sequence sample under same conditions. The conductivity cell interelectrode gap was 0.9 mm, and 9.1 mM NaOH was used

Under dynamic DIC mixing conditions, using acid injection into the DIC loaded flow stream, elution peak sequences were obtained for Au sensor direct contact (50 kHz, 100 mV rms, 0 V DC) and C4D conductivity cells, examples of which are shown in Fig. 9. These data were captured for 9.1 mM NaOH solution, as used previously.

**Fig. 10:**
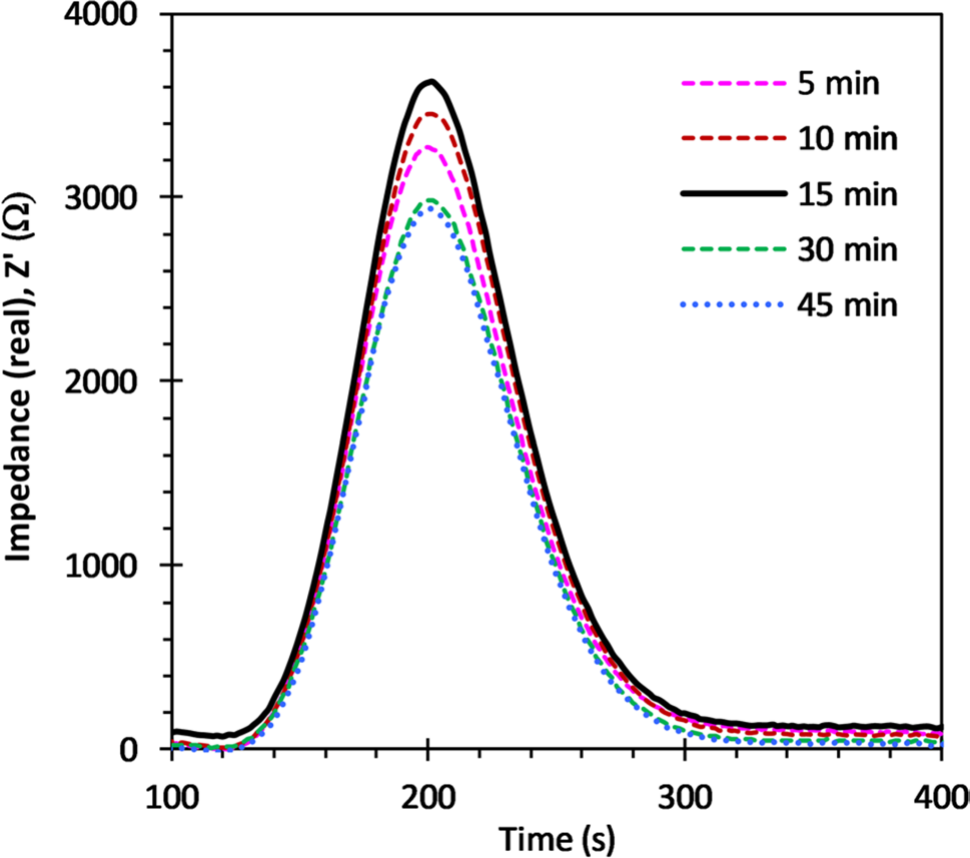
Elution peak characteristics for diffusion times up to 45 min, with 0.9 mm interelectrode gap, and7 mM NaOH receiving solution

The NaOH solution was then changed to 7.0 mM (Sayles and Eck 2009), for optimising the conductivity for best response. The elution characteristics were tested for various membrane diffusion periods from 5 min until 45 min, Fig. 10, and under low and high flow conditions, Fig. 12. The |ΔZ’| signals were found to increase substantially from ~ 2100 to ~ 3500 Ω, with the change from 9.1 to 7.0 mM NaOH, for the same 15 min diffusion hold time, and flow characteristics throughout.

**Fig. 11:**
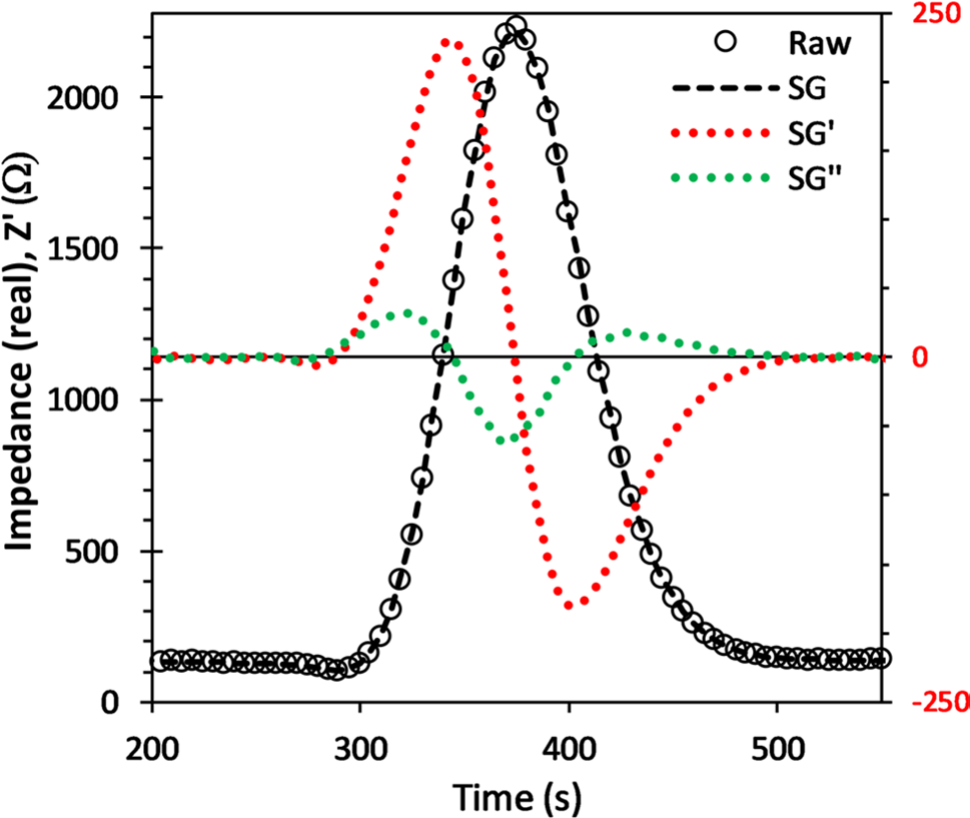
Elution peak raw data to which a Savitzky-Golay smoothing function is applied (SG). The generated 1st (SG’) and 2nd (SG’’) derivatives are used to determine start and end points, along with peak position

Measurement precision was determined from peak sequences taken over many hours with peak heights and area values extracted using an automatic procedure. First, a quadratic Savitzky-Golay smoothing function was applied to the raw signal and using the first and second Savitzky-Golay derivatives, the start, end and peak positions were obtained, Fig. 11 (Gorry 1991). The baseline represents the conductivity of the NaOH blank, the average value of which was determined using a 50 s window on either side of each peak, offset from the peak start and end points by 25 s, and subsequently subtracted from the signal. The ultimate achievable precision values, obtained from baseline SNR, are ~ 0.10% and ~ 0.15%, for Z’ and CFD, respectively. However, the uncertainty in Z’ and C4D signal peak height and area values, obtained from the long sequence measurements, depends on diffusion time and flow rate, as well as membrane instabilities, and residual temperature drifts.

**Fig. 12:**
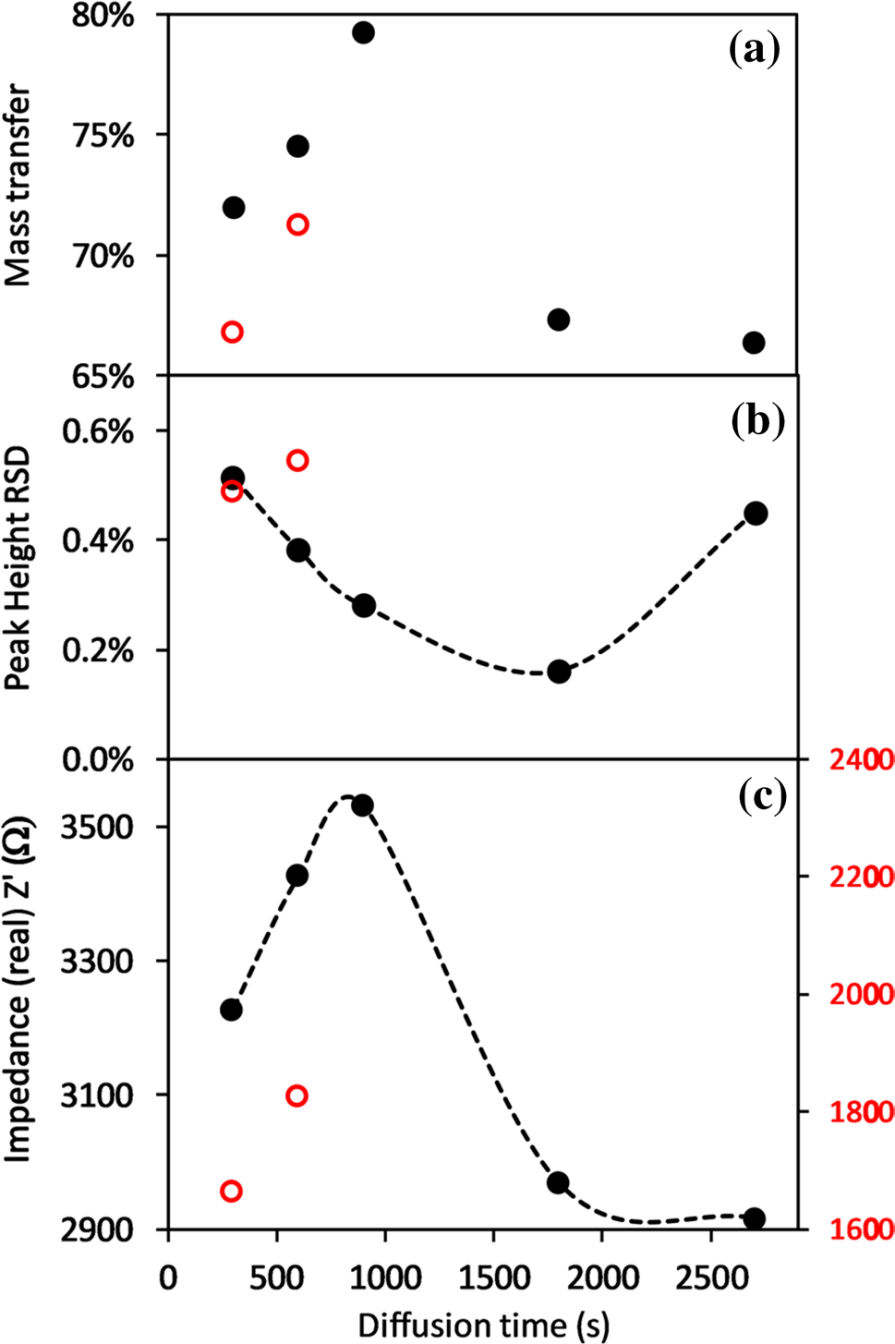
**a** Mass transfer ratio across membrane against diffusion hold time for low (solid black, 1.6 µL s^−1^) and high (red, 12 µL s^−1^) flow conditions. **b** Background-subtracted Z’ peak RSD and **c** absolute height values for 2000 µM DIC. Interelectrode gap was 0.9 mm, with 7.0 mM NaOH

The peak height and its RSD variation with diffusion time is shown in Fig. 12. The former increases with diffusion time up to 15 min and then falls thereafter, while the RSD variation is almost the inverse, decreasing to a minimum of < 0.2% for 30 min diffusion. The DIC mass transfer ratio, obtained from integrating the area under the curve, follows the same trend as peak height with a maximum value of 80% occurring after 15 min diffusion, falling to ~ 65% after 45 min. Under low flow conditions, the average pulse width varied from 210 to 228 s. This equates to a reagent volume of ~ 350 µL, for a sample input volume of 100 µL. Increasing the flow rate by a factor of 7.5–12 µL s^−1^, results in higher RSD with reduced peak heights and mass transfer ratios of around 70% or less. The reagent volume increased to > 600 µL but the pulse width reduced to ~ 50 s. The equivalent analysis using peak areas rather than heights showed similar trends, but the RSD values were higher, with a minimum of 0.5% after 15 min diffusion.

There are several potential contributing factors to the observed precision and any given condition. Some will remain intrinsic to the microfluidic device, while others may be influenced by laboratory experimental conditions. The latter include syringe precision, bench electronics and instrumental temperature fluctuation. Ocean capable fixed volume solenoid pumps e.g. custom modified Lee LPV Series, with oil fill holes for pressure equalisation in a collapsible bag, offer sufficient precision of 0.04% of the full pump stroke for this task. For a 750 µL pump, precision would be 0.3 µL. Modern precision laboratory syringes, e.g. Cetoni Low Pressure NeMeSys, can offer flow rates of as low as 0.6 nL/min with a 1 mL syringe, suggesting these may also be sufficiently precise (< 0.1% rms at ~ 10 µL/min).

The electronic noise level will depend on the number of useful measurements as N^0.5^, which in turn depends on measurement frequency, CO_2_-loaded NaOH volume and flow rate. The dedicated C4D electronics allows sampling rates of up to 100 Hz at a single excitation frequency, whereas the Solartron in this set up is limited to a maximum sampling rate to 0.2 Hz. The use of a low flow rate is limited by analyte diffusion, discussed below.

The differences in C4D results here (0.25%) compared to those reported by Bresnahan and Martz (0.1%) for a similar measurement setup, are due primarily to the use of long (165 mm) tubular membranes, in the latter case, with a high surface area-to-volume ratio. They observed precision values > 0.5% for planar cell constructions but performance improved by increasing sample volume and, to a lesser degree, diffusion time. The latter determines the equilibration fraction.

Assuming similar diffusion rates for CO_2_ in PDMS and water, the time constant (*l* = √Dt) for filling a 1 mm deep receiver chamber is approximately 500 s and hence the 80% equilibration after a 900 s exchange diffusion time, Fig. 12 is to be expected (Merkel et al. 2000; Cadogan et al. 2014). Complete equilibration therefore requires extended diffusion times or a reduction in the chamber depth. The latter option significantly increases flow resistance and hence the achievable flow rate, for given pump capability and power, and would also limit the depth sampling resolution on a float. While longer diffusion times would increase the analyte concentration in the receiver chamber, sideways diffusion along input and output channels of the receiving chamber will widen the elution peak and increase effective losses as the edge concentrations approach a limit of detection (LOD) around 20–25 µmol kg^−1^ (~ 3σ). Similar diffusion will also occur on the sample side, exacerbating this issue. To avoid the need for additional check valves either side of sample and receive chambers, we investigated the approach of using large chamber volumes with narrow inlet and outlet channels to minimise mass diffusion. However, increasing the chamber width or depth led to significant analyte trapping in dead zones and required greatly increased sample and reagent volumes for flushing between individual samples. We also observed, especially with wider channels, oscillatory flexing of the membrane into the receive chamber. This may set up pulsating flow or partial blockage and needs to be minimised or avoided altogether.

The use of microfluidic volume exchange and conductivity cells demonstrates the potential of the proposed approach for ocean chemical analysis. Known factors which affect results include thermal drift and stored reagent drift. On ocean probes, the first of these can be compensated for by analysing stored samples only at a fixed park depth. Reagent drift can be compensated for by performing regular calibrations, for low, high and mid-point DIC values.

There is a possible trade-off between analysis time (equilibration and measurement) and precision. In the case of the Argo system, probes are parked at depth between profiles for ~ 10 days, so that it may be preferred to collect samples during descent and use extended analysis times for improved precision. A full profile could collect ~ 50 samples, and with ~ 180 profiles in 5 years, at 500 µL of reagent usage per sample, would require ~ 3.5 L of NaOH and 0.1 L of HCl. To reduce the reagent storage further will require commensurate reduction of the exchange and conductivity cell volumes without loss of precision. Also, to avoid inter-sample diffusion over the extended analysis period will require development of robust ultra-small non-hydraulic microvalve networks. Some applications, however, such as instruments tethered to moored buoys, or within autonomous surface vehicles, will not place such strong constraints on sample and reagent volumes, and so facilitate easier implementation.

## Conclusions

Conductimetric determination of dissolved inorganic carbon concentration, in the seawater range of 1000–3000 µmol kg^−1^, has been achieved using a microfluidic thin-film electrode conductivity cell and a membrane-based gas exchange cell. After transfer across the membrane, the eluted CO_2_ reacted in a NaOH carrier, was drawn through a conductivity cell, with a < 1 µL interelectrode volume, where the change in impedance versus time was measured. Minimum precision values obtained at 2000 µmol kg^−1^ from relative standard deviation were ~ 0.2% from peak height and 0.5% from area under curve. This compares favourably with precision values of ~ 0.25% obtained using a large C4D electrophoresis headstage of similar active measurement volume. The required sample and reagent volumes amounted to ~ 500 µL in total due to the incorporation of a planar membrane into a small-volume exchange cell. This compares very favourably with previous attempts at conductivity-based DIC analysis where total volumes between 5000 and 10,000 µL were required while achieving precision values approaching 0.1% also necessitated the use of 20 cm long membrane tubes and wire electrodes. The achievement here of high precision miniaturisation suggests the potential for future development of a lab-on-chip-based conductimetric analysis approach for autonomous continuous measurement of ocean chemistry via float deployment, or on other platforms and measurement equipment. Performance improvement in the near future will require addressing membrane chemical and mechanical stability as well as volume reduction and component integration into a single manifold. Continuous and autonomous ocean profiling of DIC remains an immense challenge and future success is not guaranteed. The ability to measure CO_2_ to the desired precision in a microfluidic cell, as demonstrated here, is a first proof of principle of one element of any future microfluidic profiling technology.
